# FOXM1: An emerging master regulator of DNA damage response and genotoxic agent resistance

**DOI:** 10.1016/j.bbagrm.2014.09.016

**Published:** 2014-11

**Authors:** Stefania Zona, Laura Bella, Matthew J. Burton, Gabriela Nestal de Moraes, Eric W.-F. Lam

**Affiliations:** Department of Surgery and Cancer, Imperial College London, Hammersmith Hospital Campus, London W12 0NN, UK

**Keywords:** FOXM1, DNA damage, Transcriptional targets, Cancer, Drug resistance

## Abstract

FOXM1 is a transcription factor required for a wide spectrum of essential biological functions, including DNA damage repair, cell proliferation, cell cycle progression, cell renewal, cell differentiation and tissue homeostasis. Recent evidence suggests that FOXM1 also has a role in many aspects of the DNA damage response. Accordingly, FOXM1 drives the transcription of genes for DNA damage sensors, mediators, signal transducers and effectors. As a result of these functions, it plays an integral part in maintaining the integrity of the genome and so is key to the propagation of accurate genetic information to the next generation. Preserving the genetic code is a vital means of suppressing cancer and other genetic diseases. Conversely, FOXM1 is also a potent oncogenic factor that is essential for cancer initiation, progression and drug resistance. An enhanced FOXM1 DNA damage repair gene expression network can confer resistance to genotoxic agents. Developing a thorough understanding of the regulation and function of FOXM1 in DNA damage response will improve the diagnosis and treatment of diseases including cancer, neurodegenerative conditions and immunodeficiency disorders. It will also benefit cancer patients with acquired genotoxic agent resistance.

## Highlights

• FOXM1 is a potent oncogenic factor essential for cancer initiation, progression and drug resistance.
• FOXM1 also drives the transcription of genes for DNA damage sensors, mediators, signal transducers and effectors.
• It plays an integral part in maintaining the integrity of the genome.
• An enhanced FOXM1 DNA damage repair gene expression network can confer resistance to genotoxic agents.

## 1 FOXM1

Forkhead Box M1 (FOXM1), also known as HNF-3, HFH-11, MPP2, Win, and Trident, is a member the Forkhead superfamily of transcription factors [1,2]. The FOXM1 protein consists of 3 functional regions, an N-terminal Repressor Domain (NRD), a Forkhead/Winged-helix domain (FKH) and a Transcativation Domain (TAD). The NRD is found within the N-terminus and the TAD, which has transactivational activity, is located in the C-terminal. The NRD and TAD are separated by a highly conserved Forkhead DNA-binding domain (FHD). It is believed that the NRD folds back to suppress the transactivational activity of the TAD.

The human FOXM1 gene consists of 10 exons. Exons Va and VIIa can be alternatively spliced, to give rise to three common isoforms, FOXM1a, FOXM1b and FOXM1c [1,2]. FOXM1a lacks transactivation activity because of the presence of both extra exons, Va and VIIa, within its transactivation domain. Both FOXM1b (which lacks either exons) and FOXM1c (contains Va only) are transcriptionally active [1,2]. It has been suggested that FOXM1b is present in the predominant species of cancer cells and has a greater transforming potential compared with FOXM1c [3].

FOXM1 has a vital role in embryonic development, adult tissue homeostasis, cancer initiation and progression [4]. It regulates a broad spectrum of normal essential biological functions, including DNA damage repair, cell proliferation, cell cycle progression, cell renewal, cell differentiation, cell migration, angiogenesis and cell survival.

The expression of FOXM1 is frequently upregulated in many malignancies, including cancers of the liver, prostate, brain, breast, lung, colon, pancreas, skin, cervix, ovary, mouth, blood and nervous system [5–17]. Upregulation of FOXM1 expression is an early event during cancer development [13]. Accordingly, genome-wide profiling studies of gene expression in cancers have independently confirmed that FOXM1 is one of the most frequently upregulated genes in human malignancies [18,19]. These findings suggest that FOXM1 has a key role in cancer initiation. Furthermore, FOXM1 also promotes cancer progression by facilitating cancer angiogenesis, invasion and metastasis [10,20]. Recent evidence also implicates FOXM1 deregulation in the development of cancer genotoxic therapeutic agent resistance.

## 2 DNA damage and response

The genetic information of a cell is stored and encoded within its DNA, the basic building blocks of genes. During the lifespan of a eukaryotic cell, its DNA is subject to a continuous range of assaults derived from its external and cellular environment. These assaults culminate in a wide variety of DNA lesions, including nucleotide modifications and DNA breaks [21].

Agents from the external environment that induce DNA damage include ultraviolet (UV) light, ionizing radiation, toxins and genotoxic therapeutic agents. By-products of normal cellular metabolism, such as reactive oxygen species (ROS) from oxidative respiration and lipid peroxidation are amongst some of the cellular derived agents that cause DNA damage. Additionally errors made during DNA replication can also cause DNA damage. The genotoxic agents target DNA and form adducts that cause DNA lesions such as base loss, DNA single-strand breaks (SSBs) or prevent DNA replication and transcription [21]. Most of these DNA lesions will ultimately culminate in DNA double-strand breaks (DSBs), the most toxic and difficult DNA lesion to repair in cells [21].

In response to DNA damage, eukaryotic cells trigger a surveillance and reaction mechanism called DNA damage response (DDR) [21]. DDR monitors chromatin integrity, to detect and signal for the presence of DNA damage and to coordinate DNA repair with cell-cycle arrest and cell termination. Upon detection of damage, DNA damage checkpoints are activated to initiate temporary cell cycle arrest/delay [21]. Cell cycle arrest permits extra time for repair of the damaged DNA. If DNA damage is irreparable or cannot be repaired in time, DNA damage checkpoints induce cell death or senescence, a state of permanent cell cycle arrest [21,22]. Conversely, if the cell cycle checkpoints are bypassed, incorrect bases are incorporated into DNA during replication which can have adverse effects [21]. This can result in heritable mutations, which can ultimately bring about cancer and other genetic disorders. As a result, through the DDR, organisms are able to maintain integrity of the genome and prevent faulty genetic information passing on to subsequent generations of cells. Therefore, the DDR is essential for the suppression of cancer and the propagation of correct genetic information from one generation to the next.

In cancer, treatment modalities commonly comprising radiation therapy or DNA-damaging drugs, such as platinum compounds, anthracyclines, topoisomerase inhibitors and alkylation agents, are the mainstay of cancer treatment in the clinic [23–27]. These genotoxic agents are also used before surgery in neoadjuvant therapy to shrink the tumour before operation and after surgery as adjuvant therapy to prevent cancer relapse. The objective of genotoxic therapy is to induce irreparable genetic damages preferentially in the fast growing cancer cells, so that they will undergo cell death or permanent cell cycle arrest through DDR signalling, thus blocking their ability to divide and to proliferate further [28]. However, the long-term efficacy of most of these genotoxic agents is often hindered by the eventual development of resistance, which is a major cause of cancer treatment failure [29]. Recent research has revealed that FOXM1 plays a key role in DNA damaging agent resistance and if aberrantly activated or expressed may promote the development of drug resistance [30,31]. This review will discuss the emerging insights into the role of FOXM1 in the DDR, in particular evaluating its implications on cancer initiation and genotoxic agent resistance.

## 3 Modulation of the DNA damage response by FOXM1 in DNA damage repair

The central role of FOXM1 in DNA repair is underscored by the observation that increased DNA breaks are found in FOXM1-deficient cells [32]. Recent research has provided further insights into the importance of FOXM1 in the DDR. This has been shown through the use of gene expression array screens and candidate gene approaches. The ability of FOXM1 to induce DNA repair involves transcriptional control of a network of DNA damage sensing, mediating, signalling and repair genes [32–36].

In eukaryotic cells, the wide spectrum of exogenous and endogenous genotoxic agents can trigger a wide variety of DNA lesions. These lesions are managed by a broad range of DNA damage repair pathways, including nucleotide excision repair (NER), base excision repair (BER), fanconi anaemia (FA)/BRCA pathway, mismatch repair (MMR), homologous recombination (HR), non-homologous end-joining (NHEJ), and microhomology-mediated end joining (MMEJ) [21]. Double strand breaks (DSBs) are the most harmful types of DNA lesions and are predominantly repaired by HR and NHEJ [21,37]. MMEJ or Alternative end-joining (A-EJ) is a less-well-defined Ku-independent NHEJ repair pathway. It can proceed in the absence of key NHEJ factors and is highly mutagenic, often causing deletion mutations [38]. Both NHEJ and MMEJ can operate in any phase of the cell cycle but are error-prone, while HR is generally restricted to S and G2 phases. This is due to the fact that HR using sister chromatid sequences as templates to mediate accurate repair.

**Fig. 1:**
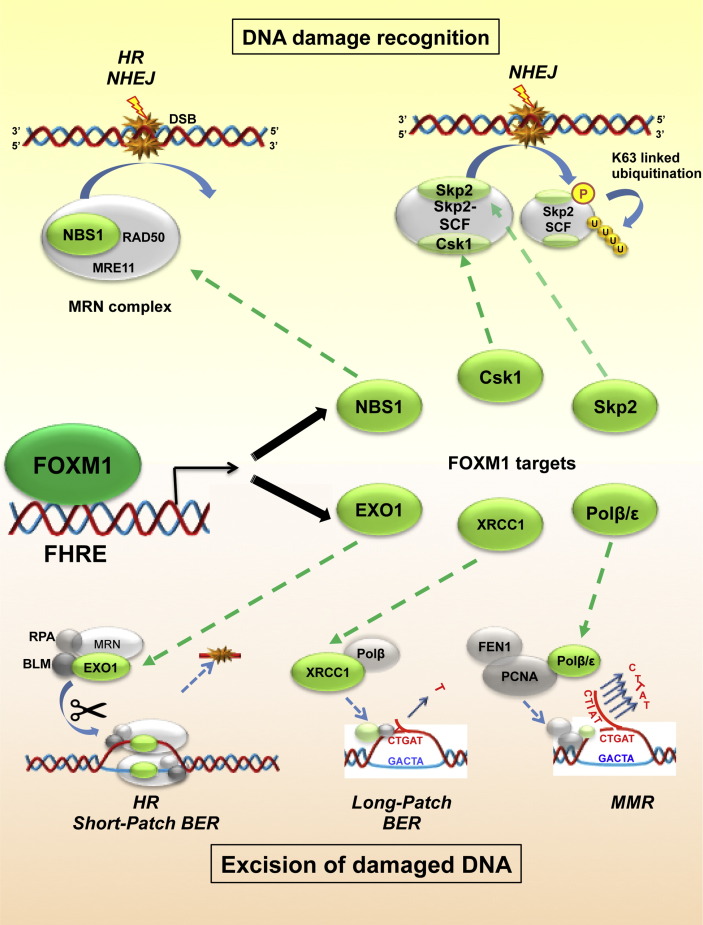
FOXM1 regulates essential mediators of DNA damage recognition and excision of damaged DNA. Schematic diagramme depicting the effect of FOXM1 binding to the Forkhead response element region (FHRE) of NBS1, Csk1, Skp2 (genes crucial for DNA damage recognition) and EXO1, XRCC1, Polβ/ɛ (excision of damaged DNA genes). All FOXM1 transcriptional targets are high-lighted in green. Clock-wise from top left corner: FOXM1 transcription of NBS1 is important for the formation of the MRN complex (NBS1, RAD50, MRE11). This participates in the recognition of double stranded DNA breaks in both homologous and non-homologous end-joining DNA damage repair processes; FOXM1 also regulates Csk1 and Skp2: these form part of the Skp2–Csk1 complex which, upon detection of double-stranded DNA damage, are phosphorylated and subsequently poly-ubiquitinated by K63, thus initiating subsequent steps in NHEJ repair; FOXM1 regulation of Polβ/ɛ permits excision of erroneous DNA sequence upon mismatch repair, when in the presence of FEN1 and PCNA; FOXM1 control of XRCC1 allows excision of the incorrect base pair when coupled with Polβ, in the long-patch base-excision repair process; FOXM1 regulates EXO1, which is key for both homologous recombination and short-patch base excision repair pathways: in combination with the MRN complex, RPA and BLM, it allows for the resection of the damaged strands.

**Fig. 2:**
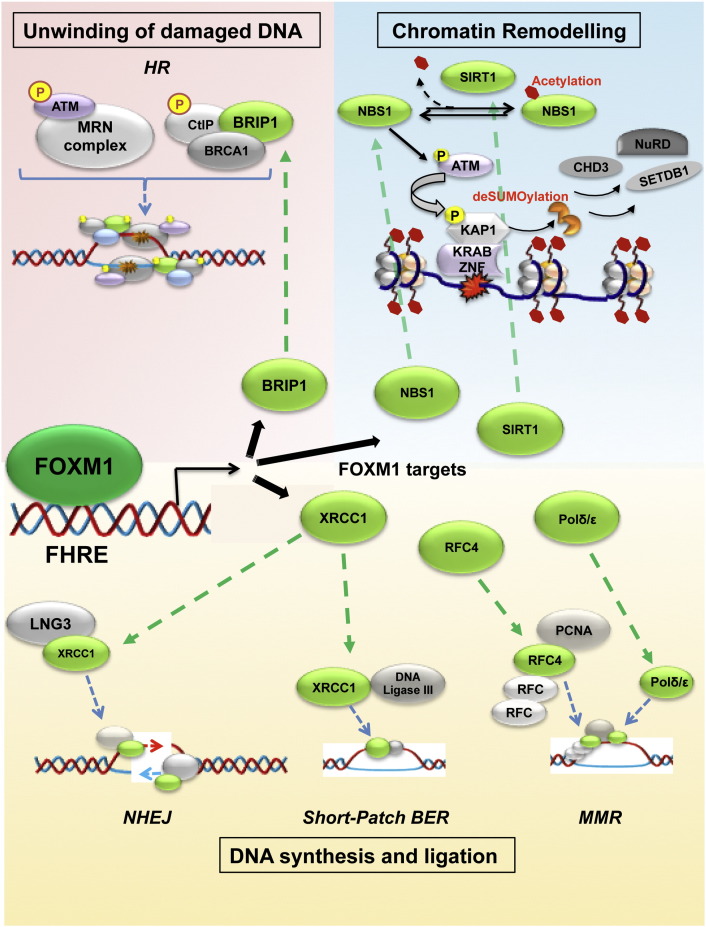
FOXM1 controls proteins that participate in the unwinding of the damaged DNA, chromatin remodelling and DNA synthesis and ligation steps of the DNA damage response pathways. Schematic diagram representing FOXM1 transcriptional control of BRIP1, NBS1, SIRT1, XRCC1, RFC4 and Polδ/ɛ and their effect on chromatin remodelling, DNA synthesis, ligation and damage unwinding. FOXM1 controls its transcriptional targets by binding to the Forkhead response elements present in their promoter regions. The targets can be distinguished by their green colour. Clock-wise from top left corner: FOXM1 transcriptional regulation of BRIP1 leads to the unwinding of the damaged DNA during homologous recombination. This step entails DNA damage recognition by the MRN complex and phosphorylated ATM, as well as the formation of the Ctlp, BRCA1, BRIP1 complex; FOXM1 controls both SIRT1 and NBS1, both critical in the chromatin remodelling prior to the initiation of the DNA damage response pathways. Upon DNA damage, SIRT1 deacetylates NBS1. NBS1 induces ATM phosphorylation, and, in turn, ATM phosphorylates KAP1, leading to its loss of SUMOylation. Subsequently CHD3, NuRD and SETDB1 are also released, allowing chromatin relaxation; FOXM1 positively regulates RFC4 and Polδ/ɛ: both participate in DNA synthesis and ligation steps with PCNA and RFC during mismatch repair; FOXM1 also controls transcription of XRCC1, which functions in DNA synthesis and ligations steps of both short-patch base-excision repair and non-homologous end-joining repair processes. To perform its action, it couples with LNG3 or DNA Ligase III respectively.

FOXM1 regulates the transcription of a multitude of genes essential for DNA damage response. NER functions to remove and replace bulky helix-distorting base lesions, such as pyrimidine dimers [21,39,40]. The correct DNA structure is restored through gap-filling and religation by replication factor C (RFC), proliferating cell nuclear antigen (PCNA), DNA polymerase (DNA pol) δ or ε, DNA ligase I and replication protein A (RPA). Out of these proteins FOXM1 transcriptionally activates the expression of DNA pol, PolE2 and RFC4, a subunit of RFC which functions cooperatively with PCNA [41]. The PolE gene encodes DNA pol ε, and mutations in this gene have recently been identified to be associated with familial adenomas and colorectal cancer (CRC) [42]. BER repairs damage to single bases caused by oxidation, alkylation, hydrolysis, or deamination throughout the cell cycle (Figs. 1 and 2) [21,43,44]. The repair is completed by nucleases (AP endonuclease), end processing enzymes (polynucleotide kinase—phosphatase), polymerases (pol β and Pol λ for short-patch BER, and pol δ and pol ε for long-patch BER) and ligases (DNA ligase III along with its cofactor XRCC1 for short-patch BER, and DNA ligase III for long-patch BER). FOXM1 is a cofactor for DNA ligase III, involved in short-patch BER [32]. In addition FOXM1 is a transcriptional regulator of the base excision repair factor X-ray cross-complementing group 1 (XRCC1) [32]. The (FA)/BRCA pathway is primarily activated by ionizing radiation, inducing inter-strand DNA crosslinks [21,45]. The (FA)/BRCA and NER repair pathways share common components, and often work together to repair single strand DNA (ssDNA) damage. FOXM1 also contributes to a number of single strand break SSB repair mechanisms by transcriptionally activating the expression of genes, such as RFC4, Exo1 and PolE2 [41]. RFC4 is a subunit of RFC, which functions cooperatively with PCNA and DNA pol. The co-operating RFC4, PCNA and DNA polymerases direct ssDNA to fill the gap left following the removal of the segment containing the mismatched base during MMR [46].

HR is a relatively error-free DSB DNA repair mechanism that uses a long homologous sequence (the undamaged sister chromatid or the homologous chromosome) to guide repair [47] (Figs. 1 and 2). In HR, DDR is initiated through the detection of DSBs by the MRN (MRE11–RAD50–NBS1) complex (Fig. 2). There is evidence to suggest that FOXM1 indirectly enhances the stability of MRN subunits, including MRE11 and RAD50, by upregulating NBS1 (Nijmegen breakage syndrome protein 1) expression at the transcriptional level [48]. Through enhanced stability of these MRN subunits, further DNA damage repair response is promoted [48]. The MRN complex helps to recruit and activate key DDR signalling kinases, including ATM at the sites of DNA damage. In turn, ATM phosphorylates H2AX, its downstream target histone, ultimately leading to the recruitment of DNA repair proteins to the damage sites [49]. In addition, the ATM kinase also directly phosphorylates modulator proteins, including p53BP1 (p53-binding protein 1), SMC1, BRCA1, NBS1 and CHK2. Such proteins are essential for triggering cell-cycle arrest, and DNA repair [50–53], and FOXM1 has been implicated in their signalling network at different levels and in many ways [32,48,50–55].

Following the detection of DNA damage, HR repair begins with nucleolytic resection of broken DNA ends facilitated by the CtBP-interacting protein (CtIP). Next, the breast cancer susceptibility gene products 1 (BRCA1), BRCA2 and several RAD51-related proteins (eg. XRCC2, XRCC3, RAD51B, RAD51C, RAD51D and DMC1), promote the displacement of RPA by the strand exchange protein RAD51, resulting in the formation of a RAD51 nucleoprotein filament [21]. FOXM1 has also been suggested to be an upstream transcriptional activator of BRCA2 [32]. BRCA2 is an important HR regulator which binds the ssDNA and directly interacts with the recombinase RAD51 to stimulate strand invasion during HR. RAD51 then searches for homologous sequences and catalyzes an exchange strand between the broken duplex and the intact sister chromatid. RAD51 itself is a direct transcriptional target of FOXM1. Induction of RAD51 by FOXM1 in glioblastomas has been shown to confer resistance to the genotoxic alkylating agent temozolomide [32].

FOXM1 directly regulates the transcription of BRCA1-interacting protein-terminal helicase 1 (BRIP1/BACH1/FRACJ) [34,56]. BRIP1 binds to and functions cooperatively with BRCA1 to promote HR repair. The BRCA1-bound BRIP1 unwinds damaged dsDNA to allow other repair proteins to access and process the damaged DNA [34,56]. The FOXM1 target BRIP1 is important in HR. This is reflected by the fact that individuals with both copies of the BRIP1 gene mutated are predisposed to the FA type J (FA-J) genetic disorder. These individuals are also prone to developing leukaemias and cancers of the head, neck, breast, stomach, ovary, cervix and skin [56–59]. These BRIP1 mutations severely reduce BRIP1 activity, resulting in DNA breaks that have not properly been repaired and genetic damage accumulating over time. Beyond HR, BRIP1 also contributes to processing interstrand crosslinks (ICLs) during MMR [21,46]. This is mediated, in a BRCA1 independent manner, through its interaction with the MutLα mismatch repair complex, consisting of the MLH1 and PMS2 heterodimer [60,61]. Upon mismatch detection, the MutS–MutL complexes direct exonuclease 1 (Exo1) to remove the segment containing the mismatched base, and Exo1 is again another FOXM1 direct target [41].

FOXM1 can further potentially enhance HR repair indirectly through promoting the transcription of S-phase kinase-associated protein 2 (Skp2) and cyclin-dependent kinases regulatory subunit 1 (Cks1) [62]. Skp2 and Cks1 are key components of the Skp2–SCF E3 ligase complex that mediates the K63-linked ubiquitination of NBS1. This process is crucial for the interaction of NBS1 with ATM, thereby the activation of ATM and its recruitment to the DNA damage foci to initiate HR repair [63]. Skp2 deficient cells consistently exhibit HR repair defects and ionizing irradiation sensitivity [63]. The MRN complex is also involved in NHEJ repair (Fig. 1), particularly in response to etoposide-induced DSBs [64]. Cells deficient in MRE11 or NBS1, but not ATM, exhibit a major NHEJ repair defect, suggesting that the function of the MRN in NHEJ repair is independent of ATM [64].

Collectively, these findings provide strong indications that FOXM1 plays an integral part in DNA damage response, driving the transcription of genes encoding for DNA damage sensors, mediators, signal transducers and effectors.

## 4 FOXM1 modulates local chromatin structure to promote DNA repair

Chromatin is the complex formed by genomic DNA and its associated proteins. The local chromatin structure controls the efficiency of DNA repair, through governing the access of DNA damage response proteins to sites of DNA damage. An example of how the chromatin structure controls DNA repair efficiency is the modulation of DDR by heterochromatin. Heterochromatin is a highly compacted chromatin that is believed to constitute an obstacle for DNA repair. The processing and repair of DSBs located within heterochromatin is much slower than those in less dense euchromatin [65]. Concordantly, DNA damage foci are formed preferentially in euchromatin compared to heterochromatin after exposure to genotoxic agents [65].

Through transcriptional control, FOXM1 directly and indirectly controls many chromatin structure-modifying genes, and thereby, plays a role in chromatin structure remodelling and DNA repair. One example in which FOXM1 directly controls chromatin structure includes its transcriptional activation of the class III histone deacetylase SIRT1. SIRT1 functions to deacetylate NBS1, thereby enabling it to be phosphorylated and so capable of activating ATM signalling [66]. At the same time, FOXM1 can promote chromatin accessibility via KAP-1 through directly promoting NBS1 expression [48] as well as enhancing its activity through inducing SIRT1 expression [66]. FOXM1 also directly regulates the transcription of the polycomb protein Bmi-1 [58], which is recruited to the sites of DNA damage and required for DNA damage-induced ubiquitination of histone H2A [67].

FOXM1 can be seen in indirect control of chromatin structure modifying genes in FOXM1-induced NBS1 activation of ATM [48]. This can indirectly facilitate the access of repair proteins at sites of DNA lesions (Fig. 2). Such ATM activation enhances DNA damage repair via (KAP-1), also known as Tripartite motif-containing 28 (TRIM28) and transcriptional intermediary factor 1β (TIF1β) [65], as ATM-mediated phosphorylation of KAP-1 can trigger chromatin relaxation at sites of DSBs [65,68,69]. FOXM1 has also been shown to regulate the expression of DNA methyltransferase DNMT1. Through DNMT1, FOXM1 plays an indirect role in chromatin remodelling at sites of DNA damage. It does this through the chromatin remodelling factor HELLS, a SNF2 (sucrose non-fermenter)-like helicase involved in promoting DNA methylation in mammalian cells [70]. Intriguingly, DNMT1 has a role in promoting DNA damage repair through decondensing chromatin at sites of DNA damage; this is independent of its methyltransferase activity [70]. It is believed that the DNMT1 DNA damage response involves a currently unknown mechanism that requires further investigation.

It is evident that FOXM1 is pertinent to DNA damage repair in ways other than just regulating the expression of crucial DNA damage sensor and repair genes. Together, these findings indicate that one such means is through promoting the expression of gene products that can modulate local chromatin structure at the damage sites to enhance DNA damage repair.

## 5 DNA damage checkpoints and FOXM1

Apart from DNA repair, the DNA damage response also impacts cell cycle progression, cell survival, cell senescence and the cellular transcription programme [71]. When damage to DNA is detected, surveillance mechanisms called ‘DNA damage checkpoints’ are activated to stall cell cycle progression, allowing extra time for DNA repair to take place. FOXM1 is an integral component of the DNA damage checkpoint signalling network. It drives the transcription of a diverse range of genes encoding for DNA damage sensors, signalling mediators and effectors for cell cycle checkpoints, cell death and senescence. The ATM/Ataxia telangiectasia and RAD3 related (ATR) and their downstream Chk1/2 kinases are key components of the cell cycle checkpoint signal transduction network. For DSBs, the DNA damage is usually detected by the MRN complex, which helps to recruit and activate ATM. ATM phosphorylates and activates downstream effectors, including p53, Chk1/2, BRCA1, to induce cell cycle arrest, transcription activation, apoptosis and senescence. In ssDNA break response, the ssDNA bound RPA complex recruits and activates the ATM–ATR protein, RAD17 and the 911 (RAD9–RAD1–Hus1) complex [72]. The activated ATR then phosphorylates both RAD17 and 911 to initiate downstream signalling required for the DNA-damage-induced cell cycle checkpoints.

Besides promoting the expression of DNA damage sensor and mediator proteins, FOXM1 also integrates the DNA damage response signals with the cell cycle machinery to engage in cell cycle checkpoints. Accordingly, the expression and transcriptional activity of FOXM1 is substantially downregulated in response to genotoxic stress through transcriptional and post-translational mechanisms. Upon genotoxic drug treatment, ATM and p53 coordinately regulate FOXM1 expression. This expression is through the E2F1 transcription factor in breast cancer cells, with ATM activating and p53 repressing FOXM1 transcription through a common E2F-site on its promoter [36,73]. In consequence, DNA damage will result in an initial induction of FOXM1 expression followed by an eventual downregulation. This occurs as p53 appears to be dominant over ATM in the regulation of FOXM1 expression. However, in the absence of functional p53, genotoxic stress will lead to an induction of FOXM1 expression through ATM and E2F1. Furthermore, the genotoxic agent epirubicin has been shown to induce FOXM1 transcription via E2F1 activating the p38 MAPK–MK2 signalling axis [73,74]. Beyond transcriptional control, the activity of FOXM1 is also fine-tuned by post-translational modifications. Previous studies have shown that treatment with DNA-damaging agents, such as γ-irradiation, etoposide and UV, promotes CHK2-induced phosphorylation of FOXM1. Such phosphorylation of FOXM1 results in the stabilization of the protein leading to the transcriptional activation of downstream DNA repair genes [32]. Recent research also suggests that this might be mediated by SUMOylation, a process that plays a part in modulating the stability of the FOXM1 protein [75]. The downregulation of FOXM1 expression through transcriptional and post-transcriptional mechanisms in response to genotoxic stress is critical for the DNA damage signals to execute the cell cycle checkpoints at G1/S, S, G2/M and M phases. This is mediated through the downregulation of cell cycle regulatory genes, such as CDC25B, PLK1, Aurora B kinase, Cyclin B1, PLK1, MYC, BUB1B and CENPF, which are under transcriptional control of FOXM1 [2]. Furthermore, there is also evidence that FOXM1 cooperates with other cell cycle gene regulators, such as B-myb and E2F, to exert wider and more comprehensive cell cycle control [76–79]. In concordance, unregulated FOXM1 expression leads to a loss in DNA damage cell cycle checkpoint control [76–79]. FOXM1 also contributes to modulating the DNA damage-induced apoptosis and senescence. It is thought that this is achieved by directly controlling the transcriptional activity of anti-apoptotic and anti-senescence genes, including Bcl-2, Survivin (BIRC5), and Bmi-1, respectively [77,80,81]. So, in summary, these findings reveal that FOXM1 impacts multiple nodes in the DNA damage checkpoint signalling network. Given the prominent role played by FOXM1 in DNA damage checkpoint control, it is likely that in response to DNA damage FOXM1 holds the balance between repair and cell termination by senescence or death.

## 6 Conclusion and future perspectives

Accumulating evidence has clearly pointed to the fundamental role that FOXM1 plays in many aspects of DNA damage response. FOXM1 drives the transcription of genes encoding DNA damage sensors, mediators, signal transducers and effectors. Individuals with impaired DDR are prone to developing cancer because of ineffective or inefficient DNA repair. Consequently, this results in the accumulation of DNA lesions and oncogenic mutations. An ineffective DDR will also result in the failure to generate immunoglobulin and T-cell receptor (TCR) diversity in B and T lymphocytes, which is essential for the recognition of pathogens and antigens. Similarly, an inadequate DDR in neurons will lead to the accumulation of DNA lesions, which is associated with neurodegenerative disorders, including ataxias, Alzheimer's, Huntington's and Parkinson's diseases. As a consequence, diminished FOXM1 activity can be involved in the pathogenesis of cancer and other DDR-related degenerative diseases, such as immune deficiencies and neurodegenerative disorders. In these circumstances where FOXM1 activity and DDR are low, it may be beneficial to induce FOXM1 activity to restore normal DDR. The central role played by FOXM1 in DNA damage response also renders it a crucial modulator of genotoxic agent resistance. FOXM1 is a promising target for therapeutic intervention to override resistance to genotoxic cancer agents, such as anthracyclines, platinum compounds and ionizing radiation. The thiazole antibiotics thiostrepton and Siomycin A are able to downregulate the mRNA and protein levels of FOXM1 and so repress transcriptional activity and induces cell death [82,83]. The fact that this effect is only seen in cancer cells, but not in non-malignant cells, indicates that cancer cells are addicted to FOXM1 overexpression [82,83]. Furthermore, breast cancer cells treated with thiostrepton also become less migratory and invasive [82]. The exact mechanism of action of both thiostrepton and Siomycin A is unclear, but could be related to their ability to bind to the FKH DNA-binding domain of FOXM1, thereby preventing FOXM1 from binding to its target genes [84]. However, targeting FOXM1 can be a double-edged sword. On the one hand, individuals with impaired DDR are predisposed to cancer and other diseases because of ineffective DNA repair. On the other hand, an enhanced DDR can confer resistance to genotoxic agents. Concordantly, it has been demonstrated that glioma cancer stem cells acquire resistance to radiotherapy through over-activation of the DNA damage checkpoint and repair response [85]. Nevertheless, an elucidation of the FOXM1 gene expression network will help to unveil the basis of cellular senescence as well as genotoxic drug resistance. This will in turn help us to devise better strategies for targeting FOXM1. The drug resistant FOXM1 gene expression network in cancer can be employed as an effective drug discovery platform to identify agents targeting DNA repair pathways as potential anticancer agents as well as chemo/radiotherapy sensitizers. The FOXM1 gene signature can also be used collectively as reliable screening, diagnostic and prognostic biomarkers for early detection of new or recurrent cancer and for predicting and monitoring genotoxic agent response.
